# Influence of carrier effect on Pd/Al_2_O_3_ for methane complete catalytic oxidation

**DOI:** 10.3389/fchem.2022.978698

**Published:** 2022-08-23

**Authors:** Shengpan Peng, Ziran Ma, Jing Ma, Hongyan Wang, Jingyun Chen, Hui Wei, Yonglong Li, Zhimin Ao, Baodong Wang

**Affiliations:** ^1^ National Institute of Clean-and-Low-Carbon Energy, Beijing, China; ^2^ School of Environmental Science and Engineering, Guangdong University of Technology, Guangzhou, China

**Keywords:** methane combustion, palladium, alumina, acid sites, carrier effect

## Abstract

Pd/Al_2_O_3_ catalysts modified by different chemical elements (Mg, Si, Ce, and Zr) were tested for methane (CH_4_) catalytic combustion, and PdO nanoparticles loaded on modified Al_2_O_3_ were systematically studied. These conditions assess the carrier effects of Pd/Al_2_O_3_ and acid strength influences on CH_4_ combustion. We observed carrier effects on activation energy through tuning Pd 3d binding energies (BEs) and on pre-exponential factors (A) through Pd dispersion and acidity on supports. When the BE of Pd 3d_5/2_ is 337.3 eV, PdO nanoparticles loaded on modified Al_2_O_3_ have excellent activity in cracking the C−H bond of CH_4_, which leads to the lowest activation energy (*E*_*a*_), regardless of the size effect of the PdO nanoparticle. Furthermore, a theoretical construction that acid sites on catalysts promote the reversible elementary step (2Pd−OH ↔ Pd−O* + Pd* + H_2_O) right shifts improving the A dependency on the quantity of exposed Pd* and Pd−O*. As a result, Al_2_O_3_, as the carrier, not only modifies the electronic characteristics and size of supported PdO nanoparticles but also participates in the reaction process *via* acid sites on the surface of Al_2_O_3_.

## Graphical Abstract

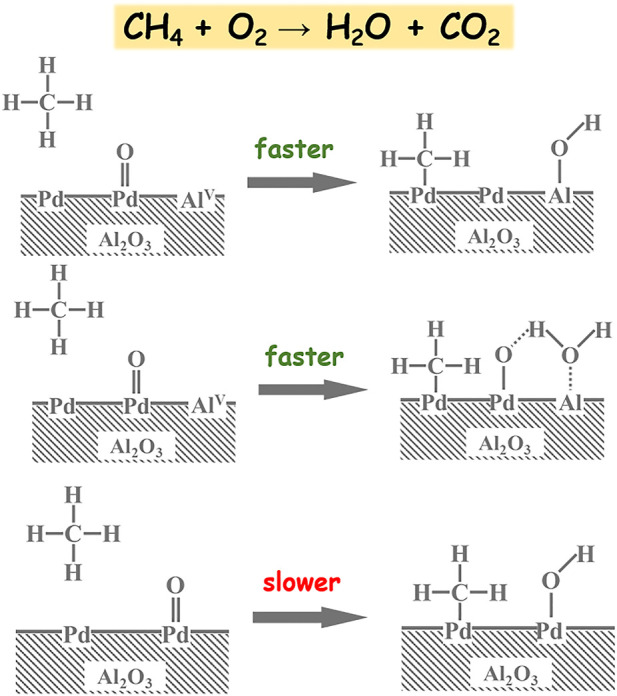

## Introduction

CH_4_, being one of the most significant greenhouse gases, has a global warming potential 20 times greater than carbon dioxide (CO_2_) (Zimmerle et al., 2020). Anthropogenic activities are to blame for the rise in atmospheric methane (CH_4_) throughout the industrial era, according to the Intergovernmental Panel on Climate Change (IPCC) Fifth Assessment Report in 2013. Anthropogenic sources account for over 60% of worldwide methane emissions, with fossil fuel production and usage accounting for roughly one-third of anthropogenic methane emissions (Alden et al., 2020). The majority of emissions from fossil fuels come from natural gas supply networks (Anthony 2008; Yang et al., 2022a). Recently, greater scientific, governmental, social, and industry focus has been placed on CH_4_ emissions (Cardoso-Saldana and Allen, 2020). The catalytic oxidation of CH_4_ has been widely investigated (Li et al., 2020; Jang et al., 2021; He et al., 2019). So far, researchers in this field have utilized a broad range of catalysts, including those based on noble metals (Pt and Pd) (Gao et al., 2021; Jang et al., 2021), transition metal oxides (Singh et al., 2020), and perovskite- and spinel-type materials (Lim et al., 2017a; Chu et al., 2022). Pd is thought to be a promising active ingredient in the oxidation of CH_4_ (Ding et al., 2020). Oxide materials, which are commonly utilized as supports for heterogeneous catalysts’ active metal nanoparticles, are known to impact catalytic activity *via* electronic metal-support interaction (Campbell 2012). The catalytic activity of supported Pd has been rationalized using aluminum oxides (Al_2_O_3_) (Gao et al., 2008; Satsuma et al., 2015a; Price et al., 2016; Shi et al., 2022), molecular sieves (Lei et al., 2020; Zhang et al., 2020), transition metal oxides (Khan et al., 2020; Li et al., 2020), and hydrotalcite oxides (Zhan et al., 2019). Al_2_O_3_ is a significant class of materials that are widely employed as catalyst supports in a variety of industries due to their large surface area and structural durability at high temperatures (Xu et al., 2021).

Extensive research has been conducted to determine how to improve the Pd/Al_2_O_3_ activity by modifying the Al_2_O_3_ surface. Chen et al. (2020a) developed uniform regular mesopore distribution and high-specific surface area Pd/Al_2_O_3_ to limit active palladium migration and sintering. Jeong et al. (2020) and Cargnello et al. (2012) create Pd@CeO_2_ structures that are uniformly anchored on the alumina support, resulting in unusually rapid methane oxidation due to improved metal-support interactions. Mg (Zhan et al., 2019), Ni (Zou et al., 2020), Co (Satsuma et al., 2015b; Lin et al., 2020), and La (Liu et al., 2013) were also used to modify the Pd/Al_2_O_3_ surface, resulting in perovskite- and spinel-type MgAlO_2_, NiAlO_2_, CoAlO_2_, and LaAlO_3_ with the maximum Pd↔PdO transformation capacity and hence high catalytic activity towards CH_4_ (Zhan et al., 2019). Notably, Yazawa et al. (1999) and Yazawa et al. (2000) found that the activity of supported Pd was influenced by the acid strength of the modified Al_2_O_3_, owing to the acidic support materials impeding the oxidation of supported palladium. However, Zhang et al. (2020) found that while alumina supports modified with and without phosphate have similar oxidation states of supported Pd, the Pd/Al_2_O_3_ without phosphate demonstrated better catalytic performance than with phosphate, indicating that formed cristobalite-type aluminum phosphate (AlPO_4_) on the Al_2_O_3_ surface decreases amounts of acid strength. Furthermore, the acid strength of the Al_2_O_3_ impacts not just the oxidation of supported Pd but may also engage in methane reaction pathways. Wang et al. (2010) verified that the unpaired electron on an O atom bonded with Al can draw a hydrogen atom from CH_4_ to produce CH3*. As a result, identifying the active species of Pd/Al_2_O_3_ catalyst and clarifying the carrier effect of Pd/Al_2_O_3_ is necessary to improve the catalytic activities.

To understand the Al_2_O_3_ carrier effects properly, we must first understand the mechanics of supported Pd catalyzing CH_4_. For decades, researchers have devoted substantial efforts to rationalizing the catalytic activity of supported Pd catalysts, and many mechanisms have been proposed (Xiong et al., 2018; Chen et al., 2020b). Monteiro et al. (2001a) found that PdO is the active phase in the reaction of methane degradation, which proved that the increase in the PdO surface area on metallic Pd foils leads to the activation rate constant of CH_4_ increasing monotonically. C−H bond activation of CH_4_ is the limit step. In PdO phase, C−H bonds can be activated by Pd^2+^-O^2−^ ion pairs, in which four-center (HC_3_^δ−^−Pd^2+^−O^2−^−H^δ+^)^⧧^ transition states formation *via* σ-bond metathesis pathways and stabilize the C−H bond (Fujimoto et al., 1998; Chin et al., 2016). Also, Pd^2+^−CH_3_^δ−^, Pd^2+^−H^δ+^, and HC_3_^δ−^−H^δ+^ interactions are stronger than with metallic Pd. Chin et al. (2016) concluded that because of the disparities in C–H bond activation transition states, activation barriers and pre-exponential variables are significantly different. Noted here, the catalytic activity varied depending on the type of support materials used. The interactions between Pd active phases and support are still not fully understood, although such information is crucial for the real application of Pd/Al_2_O_3_ in effective CH_4_ catalytic oxidation. The activity of CH_4_ catalytic oxidation of Pd/Al_2_O_3_ cannot be attributed solely to high surface area and structural stability at elevated temperature (Wang et al., 2019; Cao et al., 2023), but crystal defects of alumina, as reactive sponges (Karl et al., 1999), construct acid sites, and nanostructures affect the catalytic system (Chen et al., 2020a). For instance, Lim et al. (2017b) reported that the acidity of zeolite (Pd-SSZ-13, Pd-LTA, Pd-PST-7, and Pd-RTH) was positively correlated with the activity of CH_4_ combustion.

Herein, the carrier effect on catalytic oxidation of CH_4_ over PdO_x_ catalyst at the range of 200–500°C was studied by using a series of Al_2_O_3_ as support materials modified by Si, Mg, Ce, and Zr. In detail, 1 wt% Pd-loaded catalysts were subjected to catalytic testing under the same condition (1,000 ppm CH_4_, 10% O_2_, and N_2_ as balance gas). These data show that modified Al_2_O_3_ influences the size distribution of PdO nanoparticles, binding energy of Pd 3d, and acid strength of the catalysts. The catalytic activities of samples do not exhibit size effects consistent with the literature (Chin et al., 2016) that the smaller the PdO particles, the higher activities, which are kinetic restrictions under 700°C. The relationship between palladium oxidation state and catalytic activity in diverse combinations revealed that the activation energy for each catalyst is dependent on the palladium oxidation state. When the binding energy of Pd 3d_5/2_ is 337.3 eV, cracking the C−H bond of CH_4_, it has the lowest activation energy (*E*_*a*_). To understand the effect of Lewis acids of catalysts on pre-exponential of C–H active rates, methane oxidation of Pd-loaded pure Al_2_O_3_ has been measured. Moreover, Lewis acid sites could positively shift the reversible reaction (2Pd−OH ↔ Pd−O* + Pd* + H_2_O) into the right side, which accelerates Pd–OH species to form water and then transfers into water and surface vacancy. These processes increase the relative abundance of exposed Pd atoms (Pd*) to chemisorb oxygen molecules forming activated oxygen atoms (O*) and methane forming activated CH_4_ (CH_4_*).

## Experimental methods

### Materials

Pd(NO_3_)_2_·2H_2_O (15% as Pd) was purchased from Shanghai Jiuling Chemical CO., Ltd. Modified Al_2_O_3_ with 5 wt% Mg, 5 wt% Si, 20 wt% Ce, 20 wt% Zr, and 40 wt% Si were supported by Sasol and calcined at 600°C for 5 h. The sample names are PURAL Mg 5, SIRAL 5, PURALOX SCFa-160/Ce20, PURALOX SCFa-190 Zr20, and SIRAL 40 HPV. Al_2_O_3_ was supported by Aluminum Corporation of China and calcined at 600°C for 5 h.

### Preparation of Pd (1 wt%)/supports

200 mg of Pd(NO_3_)_2_ solution (15 wt % as Pd) is added into 5 ml distilled water (solution A). Solution A is mixed with 2.97 g of support to create a viscous slurry, which is then evaporated to near-dryness on a hot plate (while being constantly stirred) and dried for 12 h at 120°C. The final products are ground to a particle size below 100 μm and calcined in air at 550°C for 5 h using a heating ramp of 5°C/min. All Pd-loaded samples are named as 5Mg, 5Si, 20Ce, 20Zr, 40Si, and SDLY.

As comparison samples, pure Al_2_O_3_, purchased from Shanghai Aladdin Biochemical Technology Co., Ltd., was used. In addition, 1 wt% Pd was loaded on it as per the abovementioned method. Then 0.1 wt% K element (K_2_CO_3_) was impregnated on the Pd/Al_2_O_3_. The unloading K sample (named 0K) and loading sample (named 0.1K) were calcinated in air at 550°C for 5 h using a heating ramp of 5°C/min.

### Characterization techniques

A pulsed CO chemical sorption technique, which is modified from the method used by Jeong et al. (2020), is applied to determine the dispersion of palladium in the catalyst. The sample was exposed to gas flows in the following sequence: 100 mg of catalyst is heated in a 5% H_2_ flow (30 ml/min, Ar is balanced gas.) to 200°C. After cooling to 50°C, 10% CO/He is pulsed until CO chemisorption onto the catalyst is saturated. CO might be adsorbed on the ceria or zirconium surface, creating carbonates, and causing the dispersion to be exaggerated. The order in which the sample is exposed to gas flows is as follows: 100 mg of catalyst is heated to 200°C in a 5% H_2_/Ar flow with 30 ml/min. After cooling to 50°C, the sample is exposed to the following gas flows: 1) Ar (5 min); 2) CO_2_ (30 min); 3) He (60 min); and 4) 10% CO/He. Then, the mixture is pulsed until CO chemisorption onto the catalyst is saturated.

The electronic properties of Pd are studied by XPS (Al K-Alpha, Thermo Scientific, ESCALAB 250Xi). The maximum intensity of the advantageous C 1s signal at 284.8 eV is used as the reference to estimate the binding energies of the Pd element.

To get the NH_3_-TPD experiment data, 100 mg sample is heated in He flow with 30 ml/min at 200°C and extracted ab/adsorption gases and then cooled to 50°C. The sample is placed under NH_3_ atmosphere for 90 min, and then desorption at the range of 50–600°C in He flow (30 ml/min) with a ramp rate of 10°C/min. The transmission electron microscopy (TEM) apparatus JEM-ARM200F (JEOL) is used to obtain pictures of materials at an accelerating voltage of 200 kV. A small number of samples is dispersed in ethanol evenly. Then, the solution is dropped on a carbon-coated Cu grid and dried for characterization.

### Catalytic tests

The performance of the catalysts for methane oxidation was carried out in a continuous micro-reactor with an internal diameter of 13 mm at 0.1 atm. In brief,100 mg sieved catalyst grains (40–60 mesh) diluted with 400 mg quartz sand (40–60 mesh) were loaded into the reactor. The catalyst bed was held between two layers of quartz wool located in the middle of the reactor. The reactor was heated using a ceramic tubular furnace, and the temperature of the catalyst bed was controlled and monitored using a K-type thermocouple put within the reactor and close to the catalytic bed. The blank experiment loaded with quartz or catalyst supports without loading active metals was tested at different temperatures. No discernible CH_4_ conversions were observed at 300°C below.

The composition of the reactant mixture was adjusted by controlling the flow rates of CH_4_, O_2_, and N_2_, using BROOK^®^ MFC while keeping the total flow rate at a constant of 1,000 ml/min. The conditions were consistent with a gas hourly space velocity of 600,000 ml g_cat._^−1^ h^−1^. The composition of the effluent gases was analyzed by an online Fourier-transform infrared (FT-IR) gas analyzer (MKS, MultiGas™ analyzer, model 6030) equipped with a liquid-nitrogen cooled mercury-cadmium-telluride (MCT) detector installed with a ZnSe window. The detector size is 0.25 mm, and the path length is 5.11 m. The wavelength cutoff is 16 μm.

## Results and discussion

**FIGURE 1:**
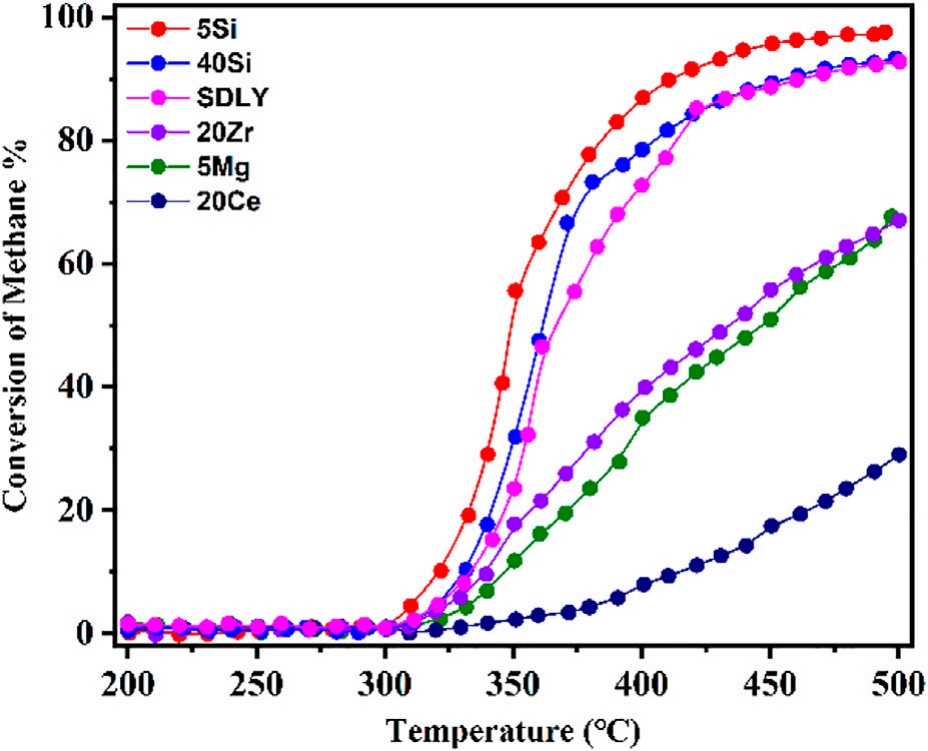
CH_4_ combustion over a series of Pd (1 wt%) catalysts at temperatures from 200 to 500°C; Reaction conditions: 0.1% CH_4_, 10% O_2_, N_2_ balance, GHSV = 600,000 ml g_cat._^−1^ h^−1^.

In this study, employing modified Al_2_O_3_ loaded with 1 wt% Pd, we investigated CH_4_ conversion on the different catalysts in the temperature range of 200–500°C at GHSV of 600,000 ml g_cat._^−1^ h^−1^with with a gas composition of 0.1% CH_4_ and 10% O_2_ balanced by N_2_, as shown in Figure 1. Keeping at the same level of Pd loading on the Pd/PURALOX SCFa-190 Zr20 (20Zr), Pd/PURALOX SCFa-160/Ce20 (20Ce), Pd/PURAL MG 5 (5Mg), Pd/SIRAL 5 (5Si), Pd/SIRAL 40 HPV (40Si), and Pd/Al_2_O_3_ (SDLY) catalysts, the derived catalysts showed very different activities, indicating that the support materials have a significant influence on the catalytic activity. The dependence of activity on the reaction temperature showed that the CH4 c conversion increased with increasing temperature, especially in the range above 350°C, but exhibited as two types of trendlines: 5Si and 40Si and SDLY showed “S” type trendlines; while 5Mg, 20Ce and 20Zr showed continuous increasing trendlines until 500°C. Among these catalysts, 5Si exhibited the best catalytic activity, whose temperatures at 10%, 50%, and 90% conversion (*T*_*10*_ *, T*_*50*_ *,* and *T*_*90*_) are 322, 350, and 412°C. 20Ce, as the most inactive catalyst, reaches 10% conversion of CH_4_ at 411°C (*T*_*10*_), 29% even at 500°C. In the temperature window of 325–400°C, the catalyst activity decreased in the sequence of 5Si > 40Si > SDLY > 20Zr > 5Mg > 20Ce which directly affects the reaction rate of methane combustion, on methane combustion.

**FIGURE 2:**
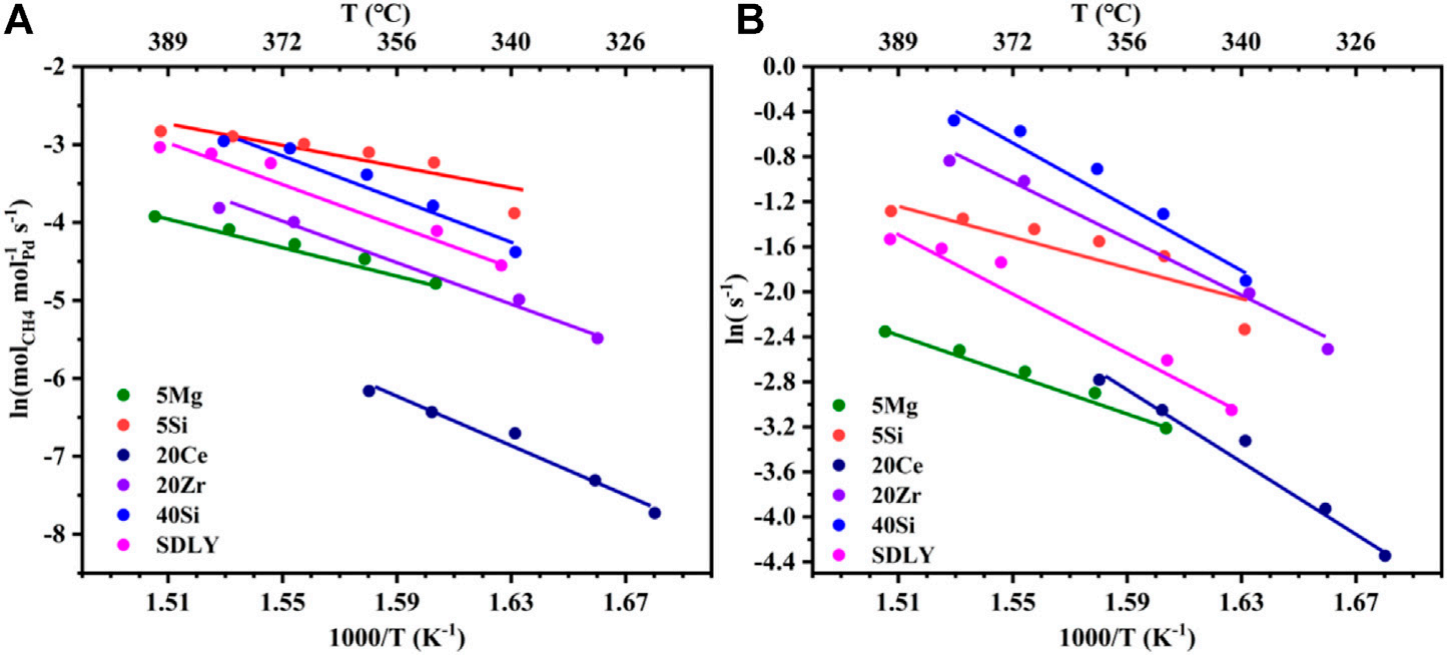
**(A)** Kinetic rate data for CH_4_ combustion on a series of Pd (1 wt%) catalysts. **(B)** Turnover frequency plot of different catalysts for the CH_4_ combustion.

A typical Arrhenius plot from which activation energies and pre-exponential factors were calculated is shown in Figure 2A. In comparison with other catalysts, the kinetic rate data on the 5Si catalyst is significantly higher than that of others, i.e., the reaction rate on the 5Si catalyst is about 20 times higher than that on 20Ce at 320°C. The apparent activation energy for each catalyst ranges from 63 to 129 kJ mol^−1^. In order to further explore the influence of the carrier effects on the intrinsic reaction rate, the TOF of a single active site as a function of the reciprocal temperature is shown in Figure 2B and Table 1. Notably, 20Zr and SDLY prepared at the same Pd loadings (1 wt%) showed very different pre-exponential factors (Ln(A)) when normalized by the number of exposed Pd atoms (Figure 2B and Table 1) and exhibited similar activation energies (105–108 kJ mol^−1^). Furthermore, the rates of 5Si are 4.5 times higher than that of 5Mg catalysts that are both exhibited the lowest activation energies (63–72 kJ mol^−1^). The data presented here demonstrate that supports play an important role in influencing the reaction kinetics, especially pre-exponential factors.

**TABLE 1:** Characteristics of Pd-loaded catalysts.

Sample	BET (m^2^/g)	Acidity (mmol/g)	Dispersion (%)^a^	Pd 3d_5/2_ (eV)	E_a_ (kJ/mol)	Ln (A′)/ln(A)^b^	TOF (s^−1^)^c^
5Mg^d^	355	1.06	20.8	337.3	72 ± 5	9.1/10.6 ± 1.0	0.008
5Si	275	1.28	21.3	337.3	63 ± 16	8.7/10.2 ± 2.9	0.034
20Ce	154	1.20	3.4	338.3	129 ± 12	18.5/21.9 ± 2.4	0.011
20Zr	195	0.73	5.1	337.0	105 ± 8	15.6/18.6 ± 1.5	0.024
40Si	472	3.30	8.4	337.6	118 ± 15	18.9/21.4 ± 2.9	0.040
SDLY	145	0.40	22.3	336.7	108 ± 11	16.7/18.2 ± 2.1	0.015
0K	131	0.034^e^	17.1	337.0	104 ± 20	18.0 ± 3.8^f^	—
0.1K	133	0.017^e^	—	337.0	104 ± 20	17.7 ± 3.8	—
0.5K	144	0.015^e^	19.8	337.0	101 ± 8	16.8 ± 1.5	—

a Dispersion (%) = 112/diameter of particles (nm).

b A′ and A are pre-exponential factors, and ln(A) = ln(A′) + ln(100/disperision).

c Turnover frequency (s^−1^) at 320°C.

d 5 Mg stands for 5 wt.% Mg in the supports, same below (SDLY and 0K are pure Al_2_O_3_ from different sources).

e Lewis acid sites are measured by pyridine with FTIR.

f Values of Ln(A).

Chin et al. (2016) and Fujimoto et al. (1998) concluded that the initial H-abstraction of methane on unsaturated Pd^2+^ sites (Pd*) on the surface of PdO particles is the rate-limiting step depending on the apparent activation energies of catalysts. The thermodynamic and kinetic barriers for the elementary steps for methane catalytic oxidation sequences depend on thermodynamic and kinetic Pd*−PdO interconversions. Monteiro et al. (2001b) studied the combustion of methane in excess oxygen over Pd and confirmed that the turnover rate and reaction orders are consistent with the equation *r* = *k*[CH_4_]^0.7^[O_2_]^0.2^[H_2_O]^−0.9^. In this study, [O_2_]/[CH_4_] is 100, and it is reasonable to assume that [O_2_]^0.2^ is a constant, which means that the chemical equilibrium of oxygen is achieved during the conversion of Pd into PdO, irrespective of the amount of methane. Because of the thermodynamical barrier with CH_4_ under a lower temperature (<350°C), the reversible reaction equilibrium of Pd→PdO shifts to the right, resulting in retardation of the rate of PdO decomposition and the nucleation of Pd* assemblies on the PdO surface continuously. Such thermodynamic barriers are overcome, which is either limited by temperature or influenced by metal-support interactions, as carrier effects tune chemical states of the surficial Pd (Pd*) on nanoparticles affecting C–H bonds activation rates in an energetically favorable path.

**FIGURE 3:**
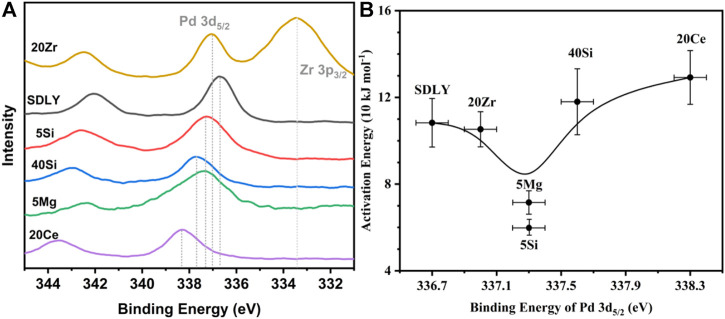
**(A)** Pd 3d XPS spectra on various supports. **(B)** Relationship between binding energy (BE) of Pd 3d_5/2_ and activation energy (*E*_*a*_).

The intrinsic endowment of Pd can be qualitative and quantified through binding energy (BE) of Pd 3d, determining the thermodynamics of Pd*−PdO interconversions in the reaction system (Chin et al., 2016; Chen et al., 2021). Price et al. (2016) reported that BE of Pd 3d on supported Pd catalysts will not change, especially the peaks values, either in an excess oxygen atmosphere or in an ultra-high vacuum without CH_4_ (Yang et al., 2022b), meaning the rationality of constructing the relationship between BE of Pd 3d in ultra-high vacuum and activation energies (*E*_*a*_), as shown in Supplementary Figure S1. First, modified Al_2_O_3_ with different species or amounts of metallic oxide leads to the change of PdO nanoparticle size and BE of Pd 3d on supports, as shown in Table 1. The Pd dispersion decreased in the sequence of SDLY ≈ 5Si ≈ 5Mg > 40Si > 20Zr > 20Ce, and consequently the PdO nanoparticle size decreased in the order of 20Ce > 20Zr > 40Si > 5Mg ≈ 5Si ≈ SDLY. It is of note that smaller PdO nanoparticles of 5Mg (5.3 nm) have a lower C–H activation rate than 40Si with larger PdO nanoparticles (13.3 nm), which is contrary to the size effects that the associated thermodynamic tendency for Pd*−PdO interconversions (turnover frequency, TOF) is lower for big Pd clusters than for smaller size structures. It is possible that Chin et al. (2016) proposed the theory that C–H activation rates are kinetically prohibitive with CH_4_ if the temperature is 700°C. Such thermodynamic restrictions of C–H bond activation under 350°C cannot follow the law of size effects. In this study, we analyzed the binding energy of Pd on different supports. It is observed that 5Si and 5Mg, having the lowest *E*_*a*_ in Figure 3B, showed prominent peaks at 337.3 eV. The prominent binding energy peaks, as shown in Supplementary Table S1, are either higher or lower than 337.3 eV, corresponding to the increased *E*_*a*_, which is a coincidence with that Pd^4+^ (Wang et al., 2010), like Pd on 20Ce with 338.3 eV, and Pd^0^ (Xiong et al., 2018; Chen et al., 2020b), like Pd on SDLY with 336.7 eV. They show inactive or lower activity for CH_4_ oxidation.

**SCHEME 1:**
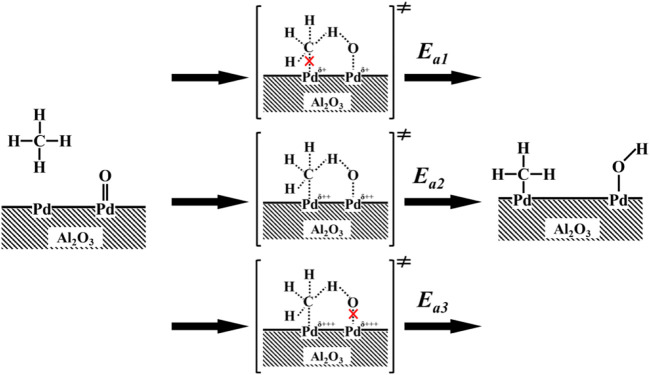
Proposed methane dissociation on a surface Pd–PdO site pair.

As Chin et al. (2016) and Fujimoto et al. (1998) proposed, in the sequence of elementary steps, unsaturated Pd sites (Pd*) and neighboring Pd–O surface species jointly and simultaneously accomplish the cleavage of C–H. Their studies show that the presence of oxygen vacancies on PdO surfaces chemisorb CH_4_ (CH_4_*) and H atoms are successively extracted from nearby Pd–O to create hydroxyl groups (Pd–OH). The recombination of surface hydroxyl groups created during C–H bond activation regenerates these vacancies in the catalytic cycle. However, the correlations between the binding energy of Pd 3d and the thermodynamics and kinetics of the Pd*−PdO interconversion remain controversial. Here we propose a mechanism to rationalize the above observation. The CH4 might dissociatively adsorb on metallic Pd and Pd–O sites, as shown in Scheme 1, the coordination of unsaturated Pd sites with positive charges (Pd^δ+^) may capture gas phase or physisorbed methane to form a four-center [H_3_C-Pd^2+^-H-O^2−^] transition states, consequently dissociate to [H_3_C-Pd^2+^] and Pd–OH]. On the catalyst with a binding energy of Pd 3d_5/2_ lower than 337.3 eV, the coordination unsaturated Pd sites (Pd^δ+^) have weak abstraction power to electron. As a result, it is hard to activate CH4 molecular and form four-center [H_3_C-Pd^2+^-H-O^2−^] transition states. When the binding energy of Pd 3d_5/2_ on the catalyst is above 337.3 eV (Pd^δ+++^), although the coordination of unsaturated Pd sites (Pd^δ+++^) has a strong capability to capture CH4 molecular, the four-center [H_3_C-Pd^2+^-H-O^2−^] transition states cannot be easily reached because of the higher barrier of formation of Pd–O–H. When the binding energy of Pd 3d_5/2_ on the catalysts is around 337.3 eV (Pd^δ++^), H atoms on dissociative absorbed CH_4_ (CH_4_*) can be abstracted immediately by neighboring Pd–O, because of the formation of the lowest activation barrier four-center [H_3_C-Pd^2+^-H-O^2−^]. Finally, the thermodynamics of Pd*−PdO interconversions can be tuned by the chemical states of Pd *via* interactions between Pd and modified Al_2_O_3_, as shown in Figure 3A, and the imperfect coordination of Pd^2+^ ions and nearby oxygen atoms has better activity in shattering the C−H bond of CH_4_, resulting in reduced methane oxidation *E*_*a*_ when the binding energy of Pd 3d5/2 is 337.3 eV (Pd^δ++^).

The acidity of catalysts is confirmed from coincided profiles of CH_4_ combustion pre-exponential factor of rate coefficient, when the *E*_*a*_ of catalysts is similar, the pre-exponential factors increase with the acid content of the catalyst. Parravano et al. (1948) reported that the exchange of C–H bonds occurs freely in a methane molecule in contact with silica–alumina catalyst at 345°C far below the cracking temperatures (700–1,000°C). But few researchers reported that modified Al_2_O_3_ without loading the active phase can degrade methane or produce another molecule (like C_2_H_6_). The contradiction can be ascribed that modified Al_2_O_3_ supports are not able to activate O_2_ in the system as O* species, and the C–C bond formation need a much higher temperature (Parravano et al., 1948). These reveal that the reaction of CH_4_ ↔ CH_3_* + H* on Al_2_O_3_ supports without an active phase is reversible. Haldeman and Emmett (1956) studied that most of the active sites of silica–alumina are Lewis acids rather than Bronsted acids for the exchange of H_2_O at a higher temperature, indicating that Lewis acids could be good for forming OH groups. To evaluate the amount of Lewis acid sites, NH_3_-TPD were chosen to measure the medium acid sites at 100–400°C (Lewis acid sites) (Gao et al., 2021), as shown in Supplementary Figure S1.

**FIGURE 4:**
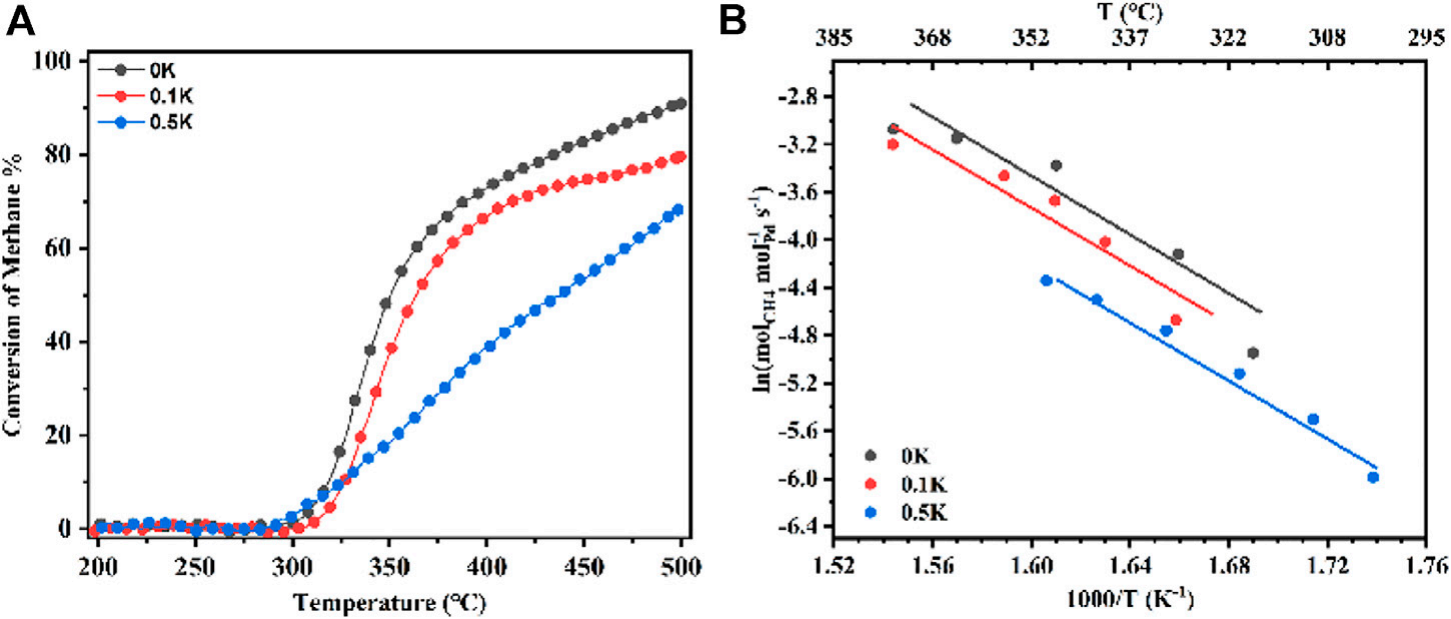
**(A)** CH_4_ combustion over a series of Pd (1 wt%) catalysts at temperatures from 200 to 500°C; Reaction conditions: 0.1% CH_4_, 10% O_2_, N_2_ balance, GHSV = 600,000 ml g_cat._^−1^ h^−1^. **(B)** Kinetic rate data for CH_4_ combustion on a series of Pd (1 wt%) catalysts. 0K means that 0 wt% K was loaded on catalysts (same as 0.1K and 0.5K).

**FIGURE 5:**
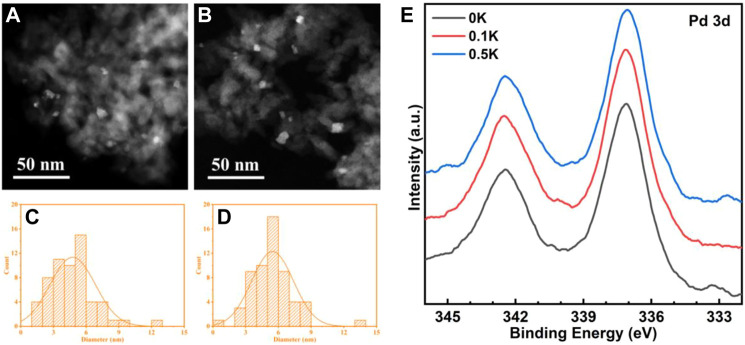
High-angle annular dark field (HAADF) micrographs of 0K **(A)** and 0.5K **(B)** catalysts; **(E)** PdO nanoparticles size distributions of 0K **(C)** and 0.5K **(D)**. **(E)** Pd 3d XPS spectra of 0K, 0.1K, and 0.5K.

To understand the effect of Lewis acids of catalysts on pre-exponential of C–H active rates, methane oxidation of Pd-loaded pure Al_2_O_3_ has been tested at the temperature range of 200–500°C as a function of the presence or absence of K_2_CO_3_ as shown in Figure 4. The samples (Figure 4) with alkali metal ions presence of 0 wt%, 0.1 wt%, and 0.5 wt% are named 0K, 0.1K, and 0.5K. As shown in Figure 4A, the catalytic activity of supported Pd (1 wt%) as a function of reaction temperature strongly depends on the support materials, in the range of 200–500°C. The catalytic activity with respect to methane conversion decreases with increasing K ions loading. Among these catalysts, 0K catalyst exhibited the best catalytic activity, corresponding to the *T*_*10*_, *T*_*50,*_ and *T*_*90*_, at 318, 350, and 499°C, respectively. Compared with 0K, the SDLY catalyst (as shown in Figure 1), the *T*_*10*_, *T*_*50,*_ and *T*_*90*_, are 333, 367, and 471°C, respectively, which showed a similar performance with that of 0K catalyst. The kinetic rate measurements indicate the 0K catalyst’s strong intrinsic activity when compared to the reference catalyst (Figure 4B). For each of the catalysts, the apparent activation energies are also similar (101–104 kJ mol^−1^) and so close to the *E*_*a*_ of 20Zr (105 kJ mol^−1^), in which the coincidence that the BEs of Pd 3d_5/2_ on 0K, 0.1K, 0.5K, and 20Zr are all same (Table 1 and Figure 5E) fitting for the curve in Figure 3B, further proved that BEs of Pd 3d_5/2_ on catalysts determine the *E*_*a*_ of methane combustion in an excess oxygen atmosphere.

The higher negative Gibbs free energy for PdO production, in which the transition of Pd↔PdO* changes O*−O* adatom sites into Pd^2+^−O^2−^ ion pairs, the smaller Pd cluster. More effective Pd^2+^−O^2−^ ion pairs for the kinetically significant C−H bond activation in CH_4_ (Chin et al., 2016). To study the influence of PdO nanoparticle size effects and dispersion on pre-exponential factors, PdO nanoparticle size distributions in 0K (Figures 5A and C) and 0.5K (Figures 5B and D) are measured, which correspond to the mean diameter of 5.8 and 5.1 nm. The 0 and 0.1K catalysts shown in Figure 5 have similar nanoparticle sizes and dispersions, indicating that 0K, 0.1K, and 0.5K have similar size effects and dispersions. Moreover, the similar specific surface area (131, 133, and 144 m^2^ g^−1^), as shown in Table 1, indicates indifferent heat and mass transfer rates because introduced K did not change the pore structure of supports. In the kinetics rate plot in Figure 4B, it is found that the pre-exponential factors A of the model catalysts (0K, 0.1K, and 0.5K) are variable as the Lewis acid contents on the catalysts are different. The pre-exponential factors increase with the increase of Lewis acid contents. In other words, the Lewis acid on the catalysts can promote the C–H activation rates by affecting the pre-exponential factor.

**SCHEME 2:**
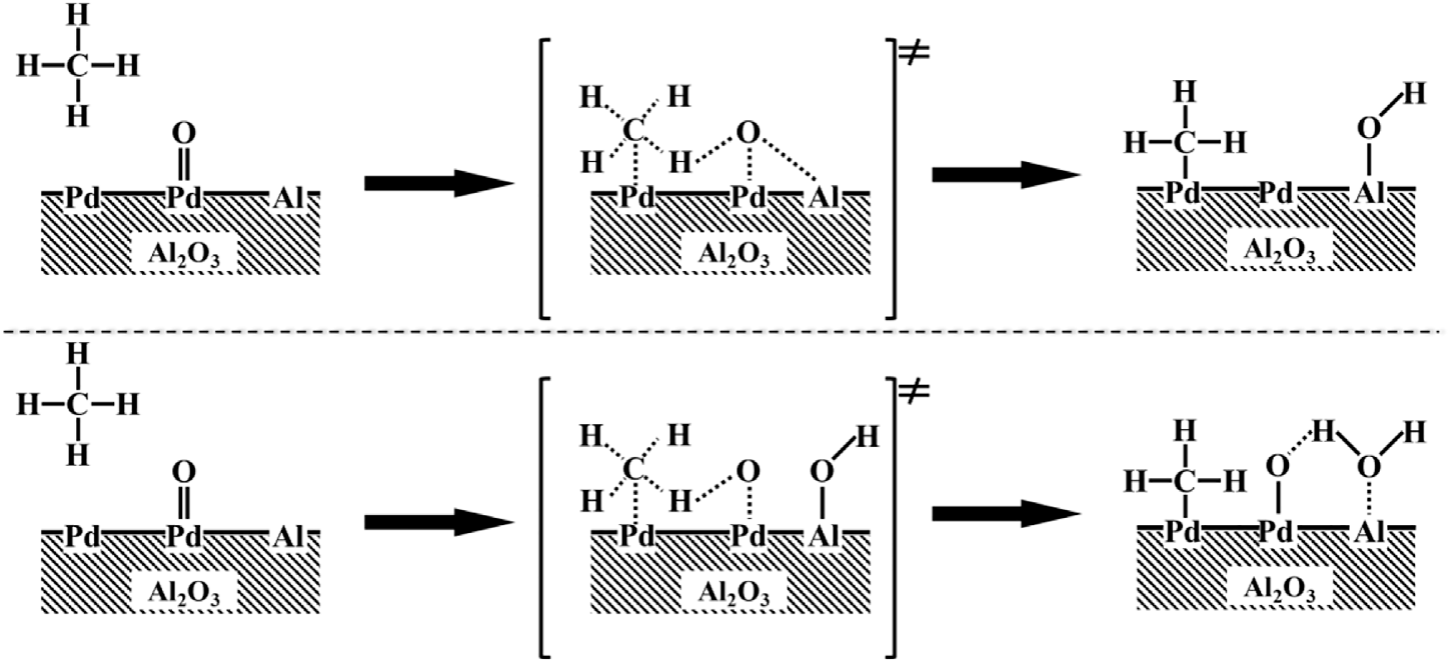
Proposed role of Lewis acid on methane dissociation on a surface Pd–PdO site pair.

The interplay between carbon atoms in the gaseous or adsorbed CH_4_ and coordinatively unsaturated Pd sites (Pd*) on the surface of PdO particles form absorbed CH_4_ (CH_4_*), according to suggested chemical pathways for the oxidation of methane on PdO (Fujimoto et al., 1998; Chin et al., 2016). Al_2_O_3_, as supports, cannot catalyze the oxidation of methane without active phases. Then the forming water molecular due to quasi-equilibrated condensation of Pd–OH species, desorption, and regenerate surface vacancies (Pd*) required for methane activation (Fujimoto et al., 1998; M. 1972). The reversible step (2Pd−OH ↔ Pd−O* + Pd* + H_2_O) determines the abundance of exposed Pd* and Pd−O*, the H-abstraction step will be blocked kinetically when Pd−OH is the most abundant surface species. Haldeman and Emmett (1956) found that hydrogen diffuses as H_2_O (OH groups or H^+^) through the Lewis acids on the support, indicating Lewis acid sites are good for the exchange of H_2_O. By assuming the inclusion of a Lewis acid site in the reaction sequence, researchers suggested the presence of Lewis acid sites near the PdO nanoparticles; these acid sites increase the abundance of exposed Pd* and Pd−O*, which induce the transfer of surface hydroxyl groups on Pd−OH to Lewis acid sites or condensation of Pd–OH and Al–OH to form water (Scheme 2). In the sequence of elementary steps of this scheme, surface vacancies (Pd*) are regenerated by the migration of hydroxyl from Pd–OH to Al because the exchange of hydroxyl between water and alumina can be measured at 100°C (Mills and Hindin, 1950), indicating the possibility of surface migration of hydroxyl between Pd–OH and Al. Parker et al. (2010) characterized the hydrous palladium oxide and found that the length of the Pd−O bond in hydrous PdO is the same as in anhydrous PdO, meaning the weakness of O−H bonds in Pd−OH and differences with OH^−^, so that O−H bonds in the Pd−OH can be easily broken. Thus, surface oxygen species (Pd–O*) can be regenerated by condensation of Pd–OH and Al–OH species to form water because of the hydrogen bond. When the amount of Pd−OH species reaches a certain constant, the quasi-equilibrated step (2Pd−OH ↔ Pd−O* + Pd* + H_2_O) results in the active sites (Pd* and Pd−O*) being continuously occupied. Al–OH/Al sites promote the positive shift in the quasi-equilibrated step so that pre-exponential factors are improved.

## Conclusion

The carrier effects on palladium oxide for methane combustion were studied in this work. The effects on activation energy were adjusted through tuning Pd 3d binding energies, and pre-exponential factors (A) were tuned by Pd dispersion and acidity on supports. First, the modification of Al_2_O_3_ by different metallic oxides can affect the interaction between metal and support, which results in a significant change in Pd 3d binding energies. The coordination of unsaturated Pd^2+^ ions (Pd*) and nearby oxygen atoms synergetically plays as the active sites for dissociation of the C−H bond of CH_4_=. When the binding energy of Pd 3d_5/2_ is at about 337.3 eV, the catalyst shows the lowest activation energy (*E*_*a*_); while increased or decreased binding energies of Pd 3d on the Pd supported different metallic oxide modified Al_2_O_3_ support. Finally, the shifts of the reversible elementary step (2Pd−OH ↔ Pd−O* + Pd* + H_2_O) depend on the density of acid sites on catalysts. The abundance of exposed Pd* and Pd−O* (improving the pre-exponential factors) appears to increase with the increase of acid strength.

## Data Availability

The original contributions presented in the study are included in the article/Supplementary Material; further inquiries can be directed to the corresponding authors.
