# Corrigendum: Influence of carrier effect on Pd/Al_2_O_3_ for methane complete catalytic oxidation

**DOI:** 10.3389/fchem.2022.1085035

**Published:** 2022-11-14

**Authors:** Shengpan Peng, Ziran Ma, Jing Ma, Hongyan Wang, Jingyun Chen, Hui Wei, Yonglong Li, Zhimin Ao, Baodong Wang

**Affiliations:** ^1^ National Institute of Clean-and-Low-Carbon Energy, Beijing, China; ^2^ School of Environmental Science and Engineering, Guangdong University of Technology, Guangzhou, China

**Keywords:** methane combustion, palladium, alumina, acid sites, carrier effect

In the original article, there was an error in the **Funding** statement, page 9, in which the funding number was incorrect. The correct **Funding** statement appears below.

“This work was supported by the Science and Technology Project of China Energy Investment, which is the development and engineering demonstration of key materials for VOCs oxidation catalysis in coal chemical industry (ST930021005C).”

The authors apologize for this error and state that this does not change the scientific conclusions of the article in any way. The original article has been updated.

