# Supplementary material for: Influence of carrier effect on Pd/Al2O3 for methane complete catalytic oxidation
Source: Front Chem. 2022 Aug 23;10:978698. doi: 10.3389/fchem.2022.978698 (PMC9445149; doi:10.3389/fchem.2022.978698)
Supplement: Supplementary file 1 [file DataSheet1.docx]

Influence of Carrier Effect on Pd/Al_2_O_3_ for Methane Complete Catalytic Oxidation

Shengpan Peng,^[a]^ Ziran Ma,^[a]^ Jing Ma,^[a]^ Hongyan Wang,^[a]^ Jingyun Chen,^[a]^ Hui Wei,^[a]^ Yonglong Li,^[a]^ Zhimin Ao,* ^[b]^ and Baodong Wang*^[a]^

^a^National Institute of Clean-and-Low-Carbon Energy, Beijing 102211, China

^b^School of Environmental Science and Engineering, Guangdong University of Technology, Guangzhou 510006, China


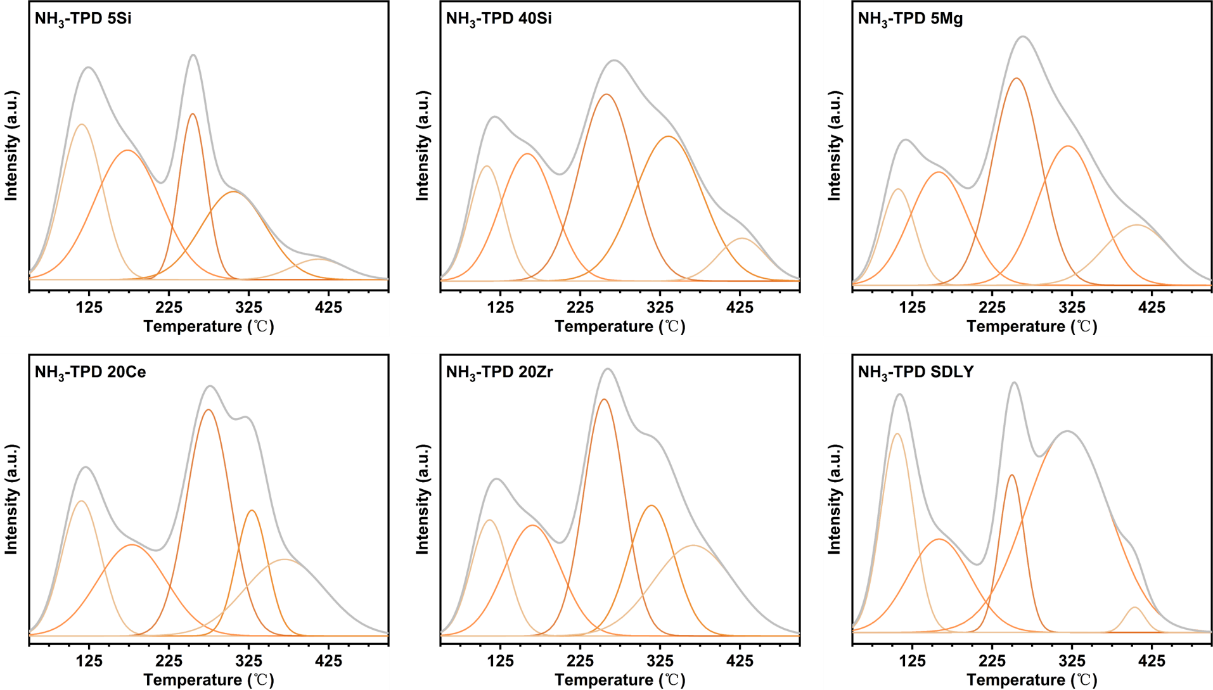


**Figure S1.** NH3-TPD of Catalysts.
